# A novel monoclonal antibody for detection of galectin-9 in tissue sections: application to human tissues infected by oncogenic viruses

**DOI:** 10.1186/1750-9378-7-16

**Published:** 2012-07-17

**Authors:** Clément Barjon, Toshiro Niki, Benjamin Vérillaud, Paule Opolon, Pierre Bedossa, Mitsuomi Hirashima, Stéphanie Blanchin, Michel Wassef, Hugo R Rosen, Anne-Sophie Jimenez, Ming Wei, Pierre Busson

**Affiliations:** 1 University Paris-Sud 11, CNRS-UMR 8126, Institut de Cancérologie Gustave Roussy, 114 rue Edouard Vaillant, 94805, Villejuif cedex, France; 2 Cellvax, Ecole Nationale Vétérinaire d’Alfort, 7 avenue du Général de Gaulle, 94704, Maisons-Alfort cedex, France; 3 Department of Immunology and Immunopathology, Faculty of Medicine, Kagawa University, Kagawa, 761-0793, Japan; 4 Département de Pathologie, INSERM U773, Hôpital Beaujon, Université Paris-Diderot, 92110, Clichy, France; 5 Px’ Therapeutics, 38040, Grenoble cedex 9, France; 6 Département de Pathologie, Hôpital Lariboisière, Université Paris-Diderot, 75475, Paris cedex 10, France; 7 Department of Medicine, Division of Gastroenterology & Hepatology, University of Colorado School of Medicine, Aurora, CO, USA

## Abstract

**Background:**

Galectin-9 is a mammalian lectin which possesses immunosuppressive properties. Excessive production of galectin-9 has been reported in two types of human virus-associated diseases chronic hepatitis C and nasopharyngeal carcinoma associated to the Epstein-Barr virus. The objective of this study was to produce new monoclonal antibodies targeting galectin-9 in order to improve its detection in clinical samples, especially on tissue sections analysed by immunohistochemistry.

**Methods:**

Hybridomas were produced through immunization of mice with the recombinant c-terminus part of galectin-9 (residues 191 to 355 of the long isoform) and semi-solid fusion of spleen cells with Sp2/0 cells. Monoclonal antibodies were characterized using ELISA, epitope mapping, western blot and immunohistochemistry.

**Results:**

We selected seven hybridomas producing antibodies reacting with our recombinant c-terminus galectin-9 in ELISA. Five of them reacted with the epitope “TPAIPPMMYPHPA” (common to all isoforms, residues 210 to 222 of the long isoform) and stained all three isoforms of galectin-9 analysed by western blot. One of them, 1G3,demonstrated very good sensitivity and specificity when used for immunohistochemistry. Using 1G3, we could confirm the intense and constant expression of galectin-9 by Epstein-Barr virus positive malignant cells from nasopharyngeal carcinomas. In most samples, specific staining was detected in both cytoplasm and nuclei. Galectin-9 was also detected in liver biopsies from patients infected by the human hepatitis C or B viruses with expression not only in inflammatory leucocytes and Kupffer cells, but also in hepatocytes. In contrast, galectin-9 was virtually absent in non-infected liver specimens.

**Conclusion:**

The 1G3 monoclonal antibody will be a powerful tool to assess galectin-9 expression and distribution especially in diseases related to oncogenic viruses.

## Background

Galectin-9 is a β-galactoside binding lectin of mammalian origin which possesses two distinct carbohydrates domains linked together by a peptide sequence of 14, 26 or 58 aminoacids depending on the isoform, respectively S, M or L isoform. Galectin-9 holds multiple immunomodulatory properties and an overall predominantly immunosuppressive function. In the context of murine immunity, galectin-9 has been shown to play a key role in a regulatory feed-back essential for a physiological termination of the Th1 immune response [1]. CD4+ Th1 lymphocytes produce interferon-gamma which induces galectin-9 production by various cell types including fibroblasts and endothelial cells. Conversely, galectin-9 induces inhibition of CD4+ Th1 lymphocytes, at least in part through stimulation of the Tim-3 receptor. It also induces expansion of regulatory T-cells in mice [2,3]. Recent studies performed in murine systems have provided novel insights about its immunosuppressive functions in the context of viral infections. In mice infected by the herpes simplex virus 1 (HSV1), galectin-9 induces apoptosis of CD4+ Th1 and CD8+ T-lymphocytes [4,5]. Interestingly these immunosuppressive effects have both adverse and beneficial effects regarding the pathological consequences of HSV1 infection. Galectin-9 favors HSV1 reactivation in the trigeminal nerve whereas it limits the extent of corneal lesions and neovascularisation in murine experimental herpetic keratitis. Galectin-9 also decreases the intensity of humoral and cellular immune response to RNA viruses like the influenza A virus in another murine experimental system [6].

Although recent data obtained in mouse experimental systems keep bringing new elements concerning the immunosuppressive and regulatory function of galectin-9, the physiological and pathological role of galectin-9 in humans remains poorly documented and controversial. There is evidence that alterations of galectin-9 functions could contribute to auto-immune diseases. For example, the Tim-3 receptor on CD4+ Th1 clones from patients with multiple sclerosis (MS) is defective in its response to galectin-9 [7,8]. Similar results were reported for patients with rheumatoid arthritis and recently auto-immune hepatitis [9,10]. Reciprocally, there is evidence of excessive galectin-9 production in two human diseases associated with oncogenic viruses : nasopharyngeal carcinomas (NPC) associated with the Epstein-Barr virus (EBV) and chronic infection by the hepatitis C virus (HCV) [11,12]. Indeed, recent works have shown the presence of tumor exosomes carrying galectin-9 in the blood of NPC patients. *In vitro*, NPC exosomes have a direct deleterious effect on several human CD4+ T-cell clones specific of EBV-antigens [11]. Using an ELISA test, we have detected unusually high concentrations of galectin-9 in the blood of patients with chronic hepatitis C, especially those HCV-related hepatocellular carcinomas [12]. It seems that in this context, galectin-9 is produced mainly by Kupffer cells. The same report demonstrates that *in vitro* recombinant galectin-9 induces expansion of regulatory T cells and apoptosis of HCV-specific cytotoxic T cells whereas it increases the production of pro-inflammatory cytokines from mononuclear cells [12]. Thus, galectin-9 may be a key element in regulating T cell response in the liver and thus in the establishment of viral persistence.

Despite the growing number of studies being published on galectin-9, no monoclonal antibody (mab) has yet been recommended for immunohistochemistry. To our knowledge, in previous publications, immunohistochemistry of galectin-9 was only based on polyclonal antibodies [13]. Therefore we have produced a collection of novel anti-galectin-9 hybridomas and we have selected one of them – the 1G3 clone - producing a mab highly efficient for staining of tissue sections. Using this antibody, we could observe strong staining of malignant epithelial cells in NPC tissue sections. We could also observe galectin-9 staining of inflammatory leucocytes, Kupffer cells and hepatocytes in liver biopsies from patients with chronic viral hepatitisC and B. This antibody is expected to become useful in a wide range of human diseases, especially those related to oncogenic viruses.

## Methods

### Production of anti-galectin-9 monoclonal antibodies

The recombinant S and M isoforms of human galectin-9 and the M isoform of murine galectin-9 were produced in E. coli as GST-fusion proteins. Tag-free proteins were purified by affinity chromatography on a lactose-agarose column [17]. The c-terminus galectin-9 (residues 191 to 355 of the galectin-9 long isoform) was produced in E. coli as a GST-fusion protein. The tag-free protein was purified by exclusion chromatography. Immunizations were conducted at PX’Therapeutics (Grenoble, France). Five BALB/c female mice (eight weeks old) were immunized with the recombinant c-terminus galectin-9. Immunizations (40 μg of protein) were administered intraperitoneally at days 0, 22, 37 and 54 with complete Freund’s adjuvant for the first immunization, then with incomplete Freund’s adjuvant for subsequent injections. We performed enzyme-linked immunosorbent assay (ELISA) on mice serum to confirm response to galectin-9 immunization, using the same recombinant galectin-9 c-terminus part which was injected into mice. A rabbit polyclonal serum raised against the same portion of galectin-9 was used a positive control. The five immunized mice exhibited a specific and strong immune response against the c-terminus part of galectin-9. Three days after the last boost, the two best responding mice were sacrificed and their splenocytes were collected to use in subsequent liquid or semi-solid fusion with Sp2/0 cells at a ratio of 5:1 and 2:1 respectively. Hybridomas supernatants were assessed in galectin-9 ELISA. The semi-solid fusion was successful and a large collection of monoclonal hybridomas secreting anti-galectin-9 antibodies was obtained.

### Galectin-9 ELISA for assessment of mouse sera and selection of hybridomas

Wells of microtiter plates (Greiner Bio-One, Courtaboeuf, France) were coated with 0.05 M carbonate/bicarbonate buffer (Sigma-Aldrich, Saint-Quentin Fallavier, France) pH 9.6 containing 50 ng human c-terminus galectin-9 during 1 H at room temperature. After washing with phosphate buffered saline (PBS) containing 0.1% Tween-20 (Euromedex, Souffelweyersheim, France), the wells were saturated with 3% bovine serum albumine (BSA) (Sigma-Aldrich, St Quentin Fallavier, France) in PBS at room temperature for 1 H. They were then incubated with mouse sera or raw hybridoma supernatants in PBS with 1% BSA at room temperature for 2 H. After a washing step with PBS with 0.1% Tween-20, the anti-galectin-9 antibody level was determined using horseradish peroxidase-coupled (HRP) goat antibodies to mouse IgG (Sigma-Aldrich, St Quentin Fallavier, France) and 3,3’,5,5’ Tetramethylbenzidine (TMB) (Thermo Fisher Scientific, Brebieres, France) as substrate. Microtiter plates were incubated 15 min in the dark under shaking before stopping the reaction with 1 M H_2_SO_4_. Then, OD was read at 405 nm and 620 nm using a MultiSkan Ex microplate reader (Thermo Fisher Scientific). Experiments were performed in duplicates.

### ELISA for epitope mapping and oligopeptide competition

A panel of 27 oligopeptides representative of the 168 amino-acids from the c-terminus galectin-9 used for mice immunisation was produced. Oligopeptides were 13 amino-acids long, with a 7 amino-acids overlap. For epitope mapping, each peptide was coated in 96-well plate using 100 ng/well during 16 hours at 4 °C. For oligopeptide competition, full length galectin-9 (recombinant S isoform produced in E. Coli) was coated in the same conditions. Wells were saturated in PBS + 0.1% BSA at room temperature during 2 hours, then incubated with purified mouse monoclonal antibodies during 2 hours at room temperature. For oligopeptide competition, 1G3 was tested in parallel with the mab 9S2-3 which targets the n-terminus of galectin-9 [14]. The antibodies were pre-incubated during 2 H at room temperature with control oligopeptide (“ITQTVIHTVQSAP”) or target oligopeptide (“TPAIPPMMYPHPA”) using serial dilutions ranging from 4 μg/ml to 2 ng/ml. Revelation of bound monoclonal antibodies was performed using a peroxidase-conjugated secondary antibody as described in the previous paragraph.

### Capture of biotinylated galectins on surface-bound antibodies

For this assay, we used the following recombinant galectins: the S and M isoforms of human galectin-9, the murine M isoform of galectin-9 and human galectin-1, -2, -3, -4, -8 (M isoform) and −10. Preparation of recombinant galectins and their biotinylation have been already described in previous works [15-17]. 1G3 and control mouse IgG1 antibody (MOPC21 clone) were coated in 96-well plates overnight at 4 °C in assay buffer (PBS-Tween, 2% fetal calf serum (FCS), 0.05% NaN_3_). Plates were blocked with 5% FCS overnight at 4 °C then washed five times with PBS-Tween. Biotinylated galectins (1 nM) were incubated during 1 H at 37 °C in antibody-coated wells; then plates were washed five times with PBS-Tween. Revelation of captured biotynilated galectins was done by addition of SA-HRP for 1 H at 37 °C. After washing, TMB substrate was added during 2.5 min at room temperature. Reaction was stopped with 1 M phosphoric acid and OD was read at 405 nm and 620 nm. Experiments were performed in duplicates.

### Cell lines

BL2 and REMB1 cells were grown in RPMI 1640 medium (Gibco-Invitrogen, Carlsbad, CA) supplemented with 10% FCS. BL2 is an EBV-negative B-cell line derived from a Burkitt’s Lymphoma. REMB1 is a lymphoblastoid cell line (LCL) resulting from *in vitro* EBV-transformation of B lymphocytes from a normal donor. HeLa cells were cultured in DMEM supplemented with 10% FCS. C15 is an EBV-positive NPC xenograft which was propagated by subcutaneous passages into nude mice [11]. C666-1 cells which are EBV-positive NPC cells were grown *in vitro* in RPMI 1640 medium supplemented with 25 mM HEPES and 7.5% FCS, in plastic flasks coated with collagen I (Biocoat; Becton-Dickinson, Franklin Lakes, NJ) [11].

### Western blot

Cell pellets were solubilized in RIPA buffer (150 mM NaCl, 25 mM Tris–HCl pH 7.5, 5 mM EDTA, 0.5% sodium deoxycholate, 0.5% NP40, 0.1% SDS) supplemented with the complete protease inhibitor cocktail (Roche Applied Science, Neuilly-sur-Seine, France) and sonicated on ice. Extracts were clarified by centrifugation for 15 minutes at 16 000 *g* at 4 °C. Protein concentration was assayed by the Bradford method using BioRad protein assay (BioRad, Marnes-la-coquette, France). Cell protein extracts were separated on 12% polyacrylamide gels in standard conditions. Gels were blotted on PVDF membranes (Immobilon-P; Millipore, Molsheim, France) then blocked during one hour with TBS containing 3% non-fat milk powder and 4% glycine. Membranes were incubated overnight with mouse monoclonal antibodies at the concentration of 2 μg/ml in blocking solution. Specific protein bands were visualized using goat anti-mouse HRP–conjugated secondary antibodies and revealed by chemiluminescence using Immobilon Western kit (Millipore, Molsheim, France).

### Clinical specimens

Thirteen samples from head and neck carcinomas were selected from a retrospective collection of biopsies collected for diagnosis purpose at Lariboisière hospital (Paris, France). This set of biopsies included ten nasopharyngeal carcinomas and three carcinomas of the oropharynx and oral cavity. Nine liver samples were selected from a retrospective collection obtained following partial or complete hepatectomy at Beaujon hospital (Paris, France). This set of surgical samples included three specimens infected by HCV, three specimens infected by the hepatitis B virus (HBV) and three specimens of uninfected patients having undergone partial hepatectomy for benign tumors. All these samples were obtained and processed according to guidelines of Lariboisière and Beaujon hospitals institutional review boards.

### Immunohistochemistry

Biopsies from head and neck carcinomas and surgical liver samples were initially fixed in paraformaldehyde 4% and paraffin-embedded. Prior to galectin-9 staining, tissue sections were dewaxed with xylene and ethanol and rehydrated. Antigens were unmasked using a pH 6.0 citrate solution at 98 °C during 30 min. After adequate washing steps, sections were incubated 10 min with 3% H_2_O_2_, washed again and saturated with blocking serum (Biogenex, MM France, Francheville, France) for 1 hour. Sections were then incubated with mouse monoclonal antibodies diluted at 2 μg/ml in blocking serum for 1 hour at room temperature. Visualization was achieved by exposing sections to a goat anti-mouse HRP 1:50 (Southern Biotech, Clinisciences, Nanterre, France) during 30 min at room temperature then adding DAB substrate. The slides were counterstained with Mayer’s hematoxylin diluted at 1:2 during 1 min at room temperature.

## Results

### Preparation and selection of hybridomas

**Figure 1:**
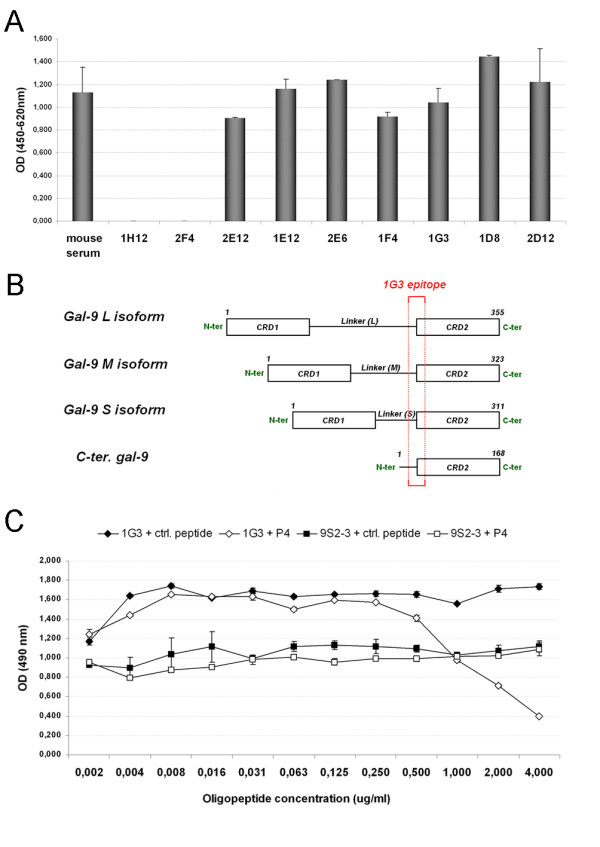
**Selection of anti-galectin 9 hybridomas and epitope mapping of the corresponding monoclonal antibodies.A**) Seven hybridomas were selected for their performance in ELISA against recombinant c-terminus galectin-9 (2E12, 1E12, 2E6, 1F4, 1G3, 1D8, 2D12). Their crude supernatants gave about the same reactivity as the undiluted mouse serum sample taken prior to spleen collection (mouse polyclonal). Other hybridomas such as 1H12 and 2F4 were set aside due to their lower reactivity with c-terminus galectin-9 in ELISA. **B**) Mapping of the 1G3 epitope (TPAIPPMMYPHPA) contained in the three natural isoforms of galectin-9. This epitope is close to the n-terminus extremity of the recombinant c-terminus galectin-9 used for mouse immunization. **C**) Selective inhibition of 1G3 binding to the recombinant S-isoform of human galectin-9 by the P4 oligopeptide (TPAIPPMMYPHPA). Plates were coated with human galectin-9 S. Subsequent binding of the mabs 1G3 and 9S2-3 were done after pre-incubation with the P4 peptide or a control peptide. The binding of 1G3 to the galectin-9 is selectively inhibited by the P4 peptide at concentrations above 0.5 μg/ml. In constrast the binding of 9S2-3 is not affected.

Five mice were immunized with the recombinant c-terminus portion of galectin-9 (residues 191 to 356 of the long isoform). Their immune response was monitored by serum ELISA on recombinant c-terminus galectin-9. As a positive control we used a previously described rabbit polyclonal serum raised against the same portion of galectin-9 [11]. Splenocytes were collected from the two best responding mice three days after the last c-terminus galectin-9 injection. Two fusions were performed, one with subsequent cloning in classical conditions (liquid medium in microwell plates) and the other in semi-solid medium. Only the fusion in semi-solid medium was successful. A panel of thirty-nine hybridomas from which culture supernatants were positive in ELISA screening was obtained. Seven were selected for their abundant secretion of immunoglobulins and high reactivity with the c-terminus galectin-9 in ELISA (Figure 1A).

### Determination of antibodies epitopes

Monoclonal antibodies produced by the seven selected hybridomas were subjected to epitope mapping, using a panel of oligopeptides (P1 to P27) representative of the 168 amino-acids from the c-terminus part of galectin-9. Five monoclonal antibodies (1G3, 1E12, 2E6, 1F4 and 1D8) were found to recognize the same linear epitope, with 1G3 consistently having the highest reactivity. The amino-acid sequence of this preferred epitope is “TPAIPPMMYPHPA” which corresponds to peptide P4 and covers the end of the linker peptide and the beginning of galectin-9 c-terminus part (Figure 1B). This sequence exists in all three isoforms of galectin-9 (aa 166 to 178 in the S-isoform, aa 178 to 190 in the M-isoform, aa 210 to 222 in the L-isoform) suggesting that those antibodies can react with all galectin-9 isoforms. Epitopes of 2E12 and 2D12 antibodies couldn’t be determined in this experiment. Therefore these antibodies may react with a conformational epitope. To confirm 1G3 specificity toward the P4 peptide, a competitive ELISA was performed using serial dilution of a control peptide and P4 peptide. 9S2-3 monoclonal antibody, which targets the n-terminus part of galectin-9, was used as control antibody [14]. As shown in Figure 1C, only the peptide P4 was able to reduce 1G3 binding to galectin-9 S isoform in a concentration-dependent manner.

### Performance in western blot

**Figure 2:**
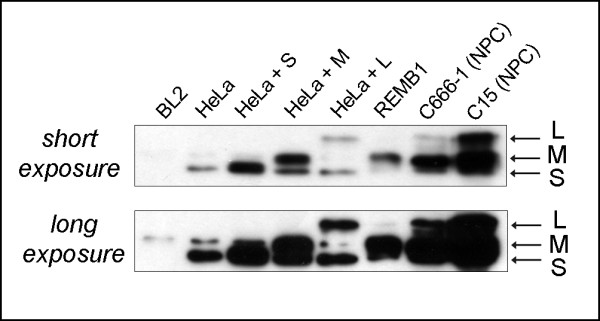
**Western blot detection of galectin-9 isoforms using the 1G3 monoclonal antibody.** A low amount of S and M galectin-9 isoforms are present in wild-type HeLa cells. These isoforms are readily detected following transfection with the appropriate expression plasmids. The L-isoform is expressed at a lower abundance than the two other forms. The three endogenous isoforms are detected in the protein extracts from the C15 and C666-1 NPC xenografts. The EBV-positive B-cell line REMB1 expresses a high amount of the M-isoform and a very low amount of L-isoform, however no S-isoform could be detected. In contrast with REMB1 cells, the BL2 cells derived from an EBV-negative B-cell lymphoma have no detectable galectin-9. The faint background bands visible after long exposure do not match with any galectin-9 isoform.

Protein extracts were prepared from various cell lines with or without endogenous expression of galectin-9. In addition, HeLa cells were short-term transfected with expression plasmids coding for each galectin-9 isoform (S, M or L). Protein extracts from each cell category were analysed by western blot using our anti-galectin-9 monoclonal antibodies. All seven antibodies were able to detect galectin-9 including 2E12 and 2D12 (data not shown). However, the strongest specific signals were consistently obtained using the 1G3 monoclonal antibody (Figure 2). All three isoforms were detected by 1G3 in HeLa cells transfected with the corresponding plasmids. A low amount of the S- and M-isoforms were detected in wild-type HeLa cells. All isoforms were detected in protein extracts from both C15 and C666-1 NPC xenografts. REMB1 cells (LCL) expressed mainly the M-isoform and a low amount of the L-isoform, while no S-isoform could be detected in this experiment. No galectin-9 was detected in BL2 cells (EBV-negative human B-cells derived from an EBV-negative Burkitt’s lymphoma) in agreement with one of our previous report [11].

### Determination of 1G3 cross-reactivity

**Figure 3:**
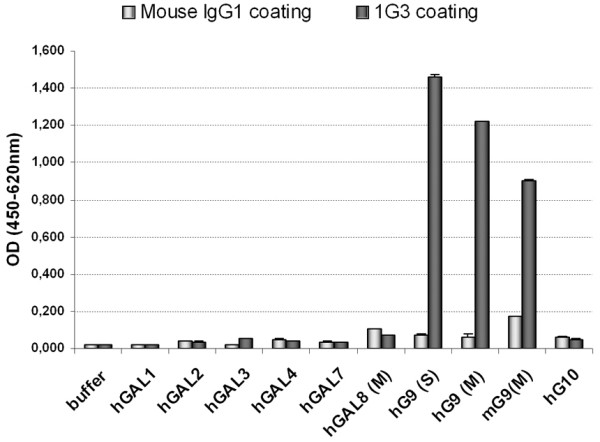
**Demonstration of the specificity of 1G3 binding to galectin-9 by a capture ELISA assay.** 1G3 and irrelevant mouse IgG were adsorbed on an ELISA plate and then incubated with various types of biotynilated human galectins (hGAL 1,2,3,4,7,8 and hG9 and hG10) or the M-isoform of murine galectin-mG9. Both human and murine galectin-9 are captured by 1G3 in contrast with other galectins which do not exhibit significant binding to 1G3.

Galectin-9 is a member of a large family of proteins which share some structural properties and conserved amino-acids. Therefore, it was important to verify that the 1G3 antibody did not cross-react with other members of the galectin family. A capture assay was performed using 1G3 or control mouse IgG bound to ELISA plates to assess their reactivity with biotynilated human galectin-1, -2, -3, -4, -8 (M isoform) and galectin-10. The M isoform of murine galectin-9 was also included in this experiment. Recombinant human galectin-9 isoforms S and M were used as positive controls. According to the data shown in Figure 3, 1G3 captures the S and M isoforms of human galectin-9 as well as the murine galectine-9. In contrast, it does not cross-react with other human galectins.

### Performance of 1G3 monoclonal antibody in immunohistochemistry

**Figure 4:**
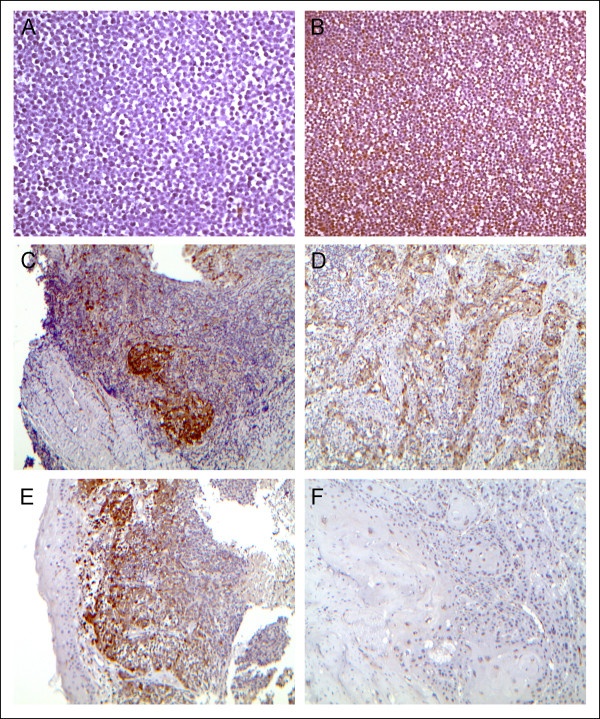
**Detection of galectin-9 in tissue sections from nasopharyngeal carcinomas and other head and neck carcinomas.** Galectin-9 is detected by immunohistochemistry using the 1G3 mab. **A**) Absence of galectin-9 staining on a section of a paraffin-embedded pellet of BL2 cells(X200). **B**) Intense albeit heterogenous galectin-9 staining on a section of a paraffin-embedded pellet of REMB1 cells (X100). **C**) NPC tumor biopsy. (Table 1, patient #2, undifferentiated non-keratinizing) (X100). Intense galectin-9 staining is seen in the nests of malignant cells which are clearly separated from the abundant lymphoid stroma. In addition, a weaker staining is seen in a large number of stromal cells. **D**) NPC tumor biopsy (Table 1, patient #10, undifferentiated non-keratinizing) (X100). Galectin-9 staining is only seen in spans of epithelial cells which are distinct from the lymphoid stroma. **E**) NPC tumor biopsy (Table 1, patient #1, undifferentiated non-keratinizing) (X100). Malignant cells are intermingled with infiltrating leucocytes. Galectin-9 is detected in both malignant and infiltrating cells. In contrast, it is completely undetectable in the non-malignant epithelium. **F**) Oropharynx squamous cell carcinoma (Table 1, patient #13) (X100). Complete absence of galectin-9 staining in malignant cells.

The seven mabs were assessed on paraffin-embedded cell pellets, using BL2 and REMB1 cells as negative and positive references, respectively. Again the 1G3 antibody was the most efficient antibody with the best signal/background ratio. It was selected for subsequent investigation of galectin-9 expression in various tissue specimens. As shown on Figure 4 and Table 1, galectin-9 expression was detected in malignant cells from all ten NPC specimens. It was consistently strong and homogeneous through the malignant cell population. Cytoplasmic staining was constant, coexisting in all but one case with nuclear staining in at least a fraction of the cells. Weaker scattered staining of stromal leucocytes was detected in six of ten cases. Tissue sections from three specimens of squamous cell carcinomas (SCC) of the oropharynx and oral cavity were also stained with 1G3 (Figure 4 and Table 1). In one of them, a tonsil SCC containing a strong lymphoid infiltrate, substantial galectin-9 staining was seen in malignant cells. In contrast, malignant cells were completely negative in tissue sections from other SCCs.

**Table 1:** Detection of galectin-9 in biopsies from head and neck carcinomas

**Patient**	**Origin**	**Gender/Age**	**Tumor site/Histological Type**	**Clinical stage**	**Galectin-9 staining**		
					**Malignant cells**	**Stroma cells**	**Adjacent mucosa**
							**(epithelium type)**
**1**	Tunisia	M/48	NPC/UNK*	T4N3bM1	**+ +**	**+**	**-** (malpighian)
**2**	Romania	F/34	NPC/UNK	T2bN0M0	**+ +**	**+**	**-** (malpighian)
**3**	Cameroon	M/62	NPC/UNK	T4N2M0	**+ +**	**-**	**+** (secretory)
**4**	France	M/63	NPC/UNK	T4N0Mx	**+ +**	**+**	**n/a**
**5**	France	M/62	NPC/UNK	T3N2M0	**+ +**	**+**	**n/a**
**6**	Portugal	F/84	NPC/UNK	T4N2M0	**+ +**	**+**	**n/a**
**7**	Algeria	M/40	NPC/UNK	T4N2M0	**+ +**	**-**	**+** (malpighian)
**8**	Turkey	M/57	NPC/UNK	T1N0M0	**+ +**	**+**	**n/a**
**9**	China	F/31	NPC/UNK	T4N0M0	**+ +**	**-**	**-** (malpighian)
**10**	France	M/48	NPC/UNK	T1N2M0	**+ +**	**-**	**-** (secretory)
**11**	France	M/62	Tonsil/Infiltrating SCC**	T2N1M0	**+**	**+**	**-** (malpighian)
**12**	France	F/82	Tongue/Differentiated SCC	T2N0M0	**-**	**+**	**-** (malpighian)
**13**	France	M/64	Pelvi-lingual furrow/Differentiated SCC	T2N1M0	**-**	**+**	**-** (malpighian)

* NPC/UNK : nasopharyngeal carcinoma/undifferentiated non-keratinizing, ** SCC : squamous cell carcinoma, ++ : intense and homogeneous galectin-9 staining, + : moderate or scattered galectin-9 staining, - : absence of galectin-9 staining, n/a : not applicable (no segment of non malignant mucosa observed in the tissue section).

For nine of these head and neck carcinoma specimens (NPC and non-NPC), fragments of non-malignant epithelium could be observed. In two cases, the epithelium was of the secretory type including one specimen with positive staining in about one third of the epithelial cells. In the seven other specimens, the epithelium was of the pluristratified Malpighian type with absence of galectin-9 staining in all but one case.

**Figure 5:**
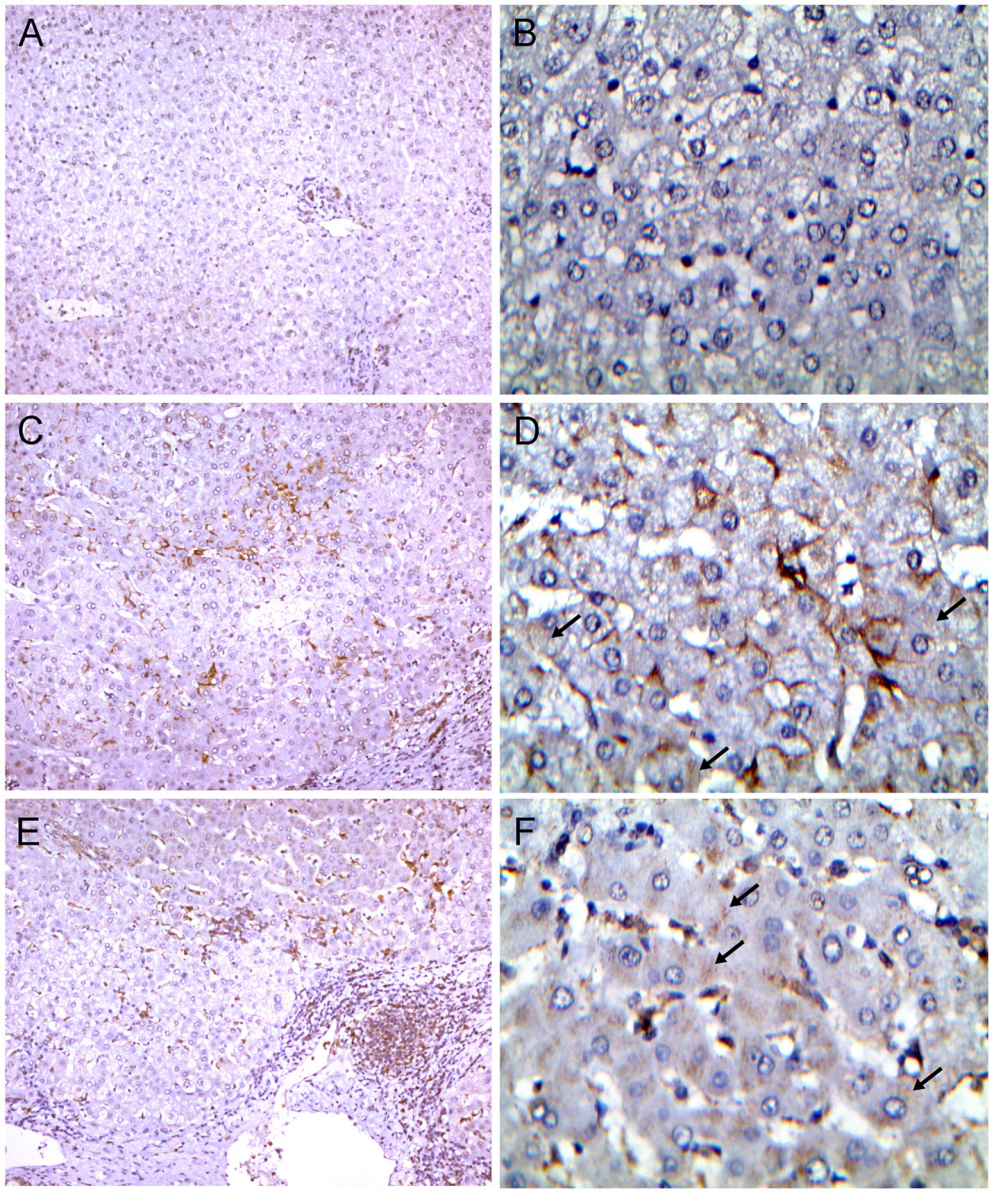
**Detection of galectin-9 in liver tissue sections.A**, **B**) Normal liver tissue next to an adenoma (Table 2, patient #8, X100 and X400 respectively). Complete absence of galectin-9 staining. **C**, **D**) HCV-related liver cirrhosis next to a carcinoma (Table 2, patient #2, X100 and X400 respectively). Prominent staining is visible in Kupffer cells which are characterized by a flat triangle shape and their close association with sinusoid vessels. At high magnification, delicate, punctate staining is visible in a large number of hepatocytes (black arrows). **E** and **F**) HBV-related liver cirrhosis next to a carcinoma. (Table 2, patient #5) Predominant staining in various types of leucocytes often with a round shape. At high magnification, delicate punctate staining is visible in a large number of hepatocytes (black arrows).

Finally, the 1G3 monoclonal antibody was tested on nine liver tissue samples infected by HCV or HBV or uninfected (Figure 5 and Table 2). Galectin-9 was consistently detected in sections from infected but not from uninfected liver specimens. At a low magnification (X10, X50 or X100), galectin-9 was not visible in hepatocytes. However it was visible in scattered polymorphic cells suggestive of inflammatory leucocytes and Kupffer cells. Restricted intense staining of cells with a morphology typical of Kupffer cells was observed in one case (patient #2) consistent with our previous report [12]. Scattered positive cells were also seen in all three specimens infected by HBV; it was not restricted to Kupffer cells but was seen in cells of various morphology probably various types of leucocytes. One additional, unexpected finding was made at a high magnification (X400). Delicate, punctate staining was visible in a large proportion of the hepatocytes in all six virus-infected liver samples. This punctate staining was almost identical for specimens infected by HCV or HBV. However it was not visible in sections from non-infected liver samples.

**Table 2:** Detection of galectin-9 in liver specimens from infected (HCV, HBV) or non-infected patients

**Patient**	**Gender/Age Medical History**	**Viral diagnosis**	**Histological diagnosis**	**Galectin-9 staining**	
				**Leucocytes or related cells**	**Hepatocytes**
**1**	M/54	Chronic HCV infection	HCV cirrhosis with transformed foci	**-**	**+ +**
	Alcoholism				
**2**	M/66	Chronic HCV infection	Micronodular cirrhosis associated to a carcinoma	**+ +** (aspect of Kupffer cells)	**+ +**
	Alcoholism				
**3**	M/61	Chronic HCV infection	Micronodular cirrhosis with transformed foci	**+**	**+**
**4**	M/67	Chronic HBV infection	Macronodular cirrhosis associated to a carcinoma	**+**	**+**
**5**	M/50	Chronic HBV infection	Hepatic fibrosis associated to a carcinoma	**+ +**	**+ +**
**6**	M/30	Chronic HBV infection	Liver tissue with minimal lesions next to a non-malignant nodule (focal nodular hyperplasia)	**+**	**+ +**
**7**	F/32	No infection	Normal liver tissue next to a non-malignant nodule (focal nodular hyperplasia)	**-**	**-**
**8**	F/20	No infection	Normal liver tissue next to an adenoma	**-**	**-**
**9**	F/28	No infection	Normal liver tissue next to an adenoma (with steatosis)	**-**	**-**

++ : homogeneous galectin-9 staining, + : mild or scattered galectin-9 staining, - : absence of galectin-9 staining.

## Discussion

Galectin-9 has complex immunomodulatory properties with action on effector cells of both innate and adaptative immunity. It induces secretion of pro-Th1 (interferon-gamma) and inflammatory cytokines by monocytes and NK-cells whereas it has an inhibitory effect on NK-cell cytotoxicity [18,19]. On the other hand it is inhibitory for mature CD4+ Th1 cells whereas it favors expansion of regulatory T-cells [1,12]. Overall galectin-9 appears to have pro-inflammatory and immuno-suppressive functions. This is consistent with its role of facilitation for various types of viral infections in murine experimental systems [4,6]. Out of seven monoclonal antibodies directed to the c-terminus of galectin-9, 1G3 was the best suited for immunohistochemistry. The epitope mapping and competition assay provide evidence that its target epitope is included in the following amino-acid sequence TPAIPPMMYPHPA (residues 210 to 222 of the long isoform). The ELISA capture assay demonstrates that it cross-reacts with the murine galectin-9. 1G3 also reacts with murine galectin-9 analysed by western blotting (data not shown). This is not surprising because murine galectin-9 contains in its linker domain an amino-acid sequence which is highly homologous to its human target (residues 208 to 220 of murine galectin-9 long isoform, “TPGIPPVVYPTPA”). In contrast, 1G3 does not react with human galectin-1, 2, 3, 4, 8 and 10 in the same assay.

Using 1G3 to analyse clinical specimens, we have confirmed that galectin-9 is abundant in two types of human tissues infected by oncogenic viruses, nasopharyngeal carcinoma and liver chronically infected by HCV or HBV. Galectin-9 was detected in malignant cells from all nasopharyngeal carcinoma specimens. Sporadic staining was also seen in some tumor infiltrating leucocytes. These finding are consistent with our initial report on galectin-9 in NPC [20]. However, in contrast with this initial report, galectin-9 was rarely detected in non-malignant mucosa. This is probably due to the fact that the polyclonal antibody used for this previous study was causing more background staining than the purified 1G3 monoclonal antibody. Galectin-9 nuclear staining was observed at least in a fraction of the cells in all but one NPC case. Nuclear distribution of galectin-9 has been recently reported in a completely different setting, precisely in microglia/macrophages of active lesions of MS stained with a rabbit polyclonal anti-galectin-9 [13]. In contrast, in chronic inactive MS lesions, galectin-9 was entirely localized in the cytoplasm. To our knowledge, a specific role for nuclear galectin-9 has not been yet described. Keeping in mind the immunosuppressive functions of galectin-9, it will be interesting to know whether relative galectin-9 abundance will be predictive of NPC tumor response to various therapeutic modalities especially adoptive immunotherapy [21,22].

Staining galectin-9 in liver specimens has resulted in confirming results and some unexpected findings. First, we have confirmed that galectin-9 is undetectable or at very low abundance in non-infected liver parenchyma. In contrast, it is detectable in HCV-infected liver tissue sections. It is noteworthy that it is also detectable in liver samples infected by HBV. In addition to Kupffer cells, it seems to be often expressed by various types of inflammatory leucocytes. Surprisingly, it is also detected in a large proportion of hepatocytes in all infected samples under the form of small punctuations localized in the cytoplasm and visible only at high magnification (X400). This means that although the amount of galectin-9 per cell is relatively low in hepatocytes, altogether these hepatocytes probably represent a major source of galectin-9 for the organisms of HCV- or HBV-infected individuals. These observations raise a series of questions to be addressed in future experiments. It will be interesting to know whether the plasma concentration of galectin-9 is increased for patients chronically infected by HBV as reported by us for patients infected by HCV [12]. Another question will be to determine to what extent the intensity of galectin-9 expression in chronically infected livers correlates with the severity of tissue lesions and the risk of hepatocarcinoma. One may also wonder whether galectin-9 abundance in tissue sections correlates with its concentration in peripheral blood.

## Conclusion

We report the performances of 1G3 a novel mab useful to assess not only the abundance and tissue distribution of galectin-9, but also its cytoplasmic and nuclear distribution. Future aims will be to investigate whether galectin-9 abundance in tissue sections is predictive of responses to various tumor modalities, especially adoptive immunotherapy. Regarding chronic hepatitis B and C, it will be useful to detemine whether galectin-9 abundance correlates with disease severity and correlates with the risk of hepatocarcinoma.

## Abbreviations

BSA, Bovine serum albumine; EBV, Epstein-Barr virus; ELISA, Enzyme-linked immunosorbent assay; FCS, Fetal calf serum; HBV, Hepatitis B virus; HCV, Hepatitis C virus; HRP, Horseradish peroxydase; HSV1, Herpes simplex virus 1; LCL, Lymphoblastoid cell line; mab, Monoclonal antibody; MS, Multiple sclerosis; NPC, Nasopharyngeal carcinoma; PBS, Phosphate buffered saline; SCC, Squamous cell carcinoma; Tim-3, T cell Ig and Mucin domain 3; TMB, 3,3’,5,5’-Tetramethylbenzidine.

## Competing interests

The authors declare that they have no competing interests.

## Authors’ contributions

CB performed characterization of monoclonal antibodies by ELISA, western blotting, epitope mapping and competitive ELISA. TN has provided the gal-9-CT plasmid construct and the polyclonal gal-9-CT antibody and has performed biotinylated galectins ELISA. SB has supervised the production of hybridomas. BV and ASJ have provided tumor cell protein extracts. MWa, PBe and BV have provided pathological samples for immunohistochemistry. PO has supervised the procedures of immunohistochemistry. PO, CB and PBu have analysed and interpreted stained tissue sections. MWe, HR and MH were involved in the design of the study and preparation of the manuscript. CB and PBu designed and coordinated the study, and drafted the manuscript. All authors read and approved the final manuscript.
